# Environmental Disease: Ozone: Good, Bad, or Indifferent?

**Published:** 2006-09

**Authors:** Bob Weinhold

Following up on their eye-catching finding that the human body generates its own ozone for beneficial purposes, a team of U.S. and British researchers now describe specific processes through which ozone can react with cholesterol and contribute to atherosclerosis, or hardening of the arteries. Whether the ozone involved comes from within the body or from the environment remains unclear, however, and the team’s findings remain controversial on several counts.

In earlier work, Paul Wentworth, Jr., a chemistry professor at The Scripps Research Institute, and colleagues concluded that self-generated ozone is used by the immune system’s antibodies and neutrophils to destroy bacteria and fungi. They published a study in 2003 showing that ozone can damage the vascular system by contributing to atherosclerosis. They also noted the same process may play a role in diseases such as lupus, multiple sclerosis, and rheumatoid arthritis.

The mechanism by which such damage occurs wasn’t clear in the 2003 study, however. Some of that information was filled in with a report published 13 June 2006 in *Biochemistry*. Through a series of *in vitro* tests, the team exposed human and mouse cells to two by-products of ozone’s interaction with cholesterol, atheronal-A and atheronal-B. They found that one, the other, or both atheronals accelerate the normal conversion of monocytes to macrophages, are rapidly taken up by macrophages, hasten the inflammatory response on and increase the stickiness of the interior arterial walls, and contribute to the formation of arterial plaques.

Numerous questions remain about the research protocols (such as a lack of controls to prove that ozone was the oxidant at work) and conclusions regarding the body’s self-generation of ozone (such as whether cells are likely to expend so much energy to produce their own ozone), says William Pryor, director of the Biodynamics Institute at Louisiana State University. Further, there is little solid evidence that environmental ozone plays a role in this specific process, although exposure to atmospheric ozone has been implicated in a number of cardiovascular problems, including heart attack and changes in heart rhythm.

Wentworth acknowledges that increased cholesterol ingestion may be the most important driving factor in the atherosclerotic damage his team found—many of the harmful atheronal processes occurred only when “bad” LDL cholesterol was present. Still, this line of inquiry may contribute to better insights about the complex relationships between the body’s normal functions, reactive oxygen species including ozone, and cardiovascular damage, leading even its critics to say this concept deserves attention, analysis, and more research.

